# Effective involvement: a report on the evaluation of a research awareness training package for public involvement in health research

**DOI:** 10.1186/s40900-019-0151-5

**Published:** 2019-06-13

**Authors:** Catherine Richardson, Ilyas Akhtar, Christine Smith, Amanda Edmondson, Alison Morris, Janet Hargreaves, Christine Rhodes, Jo Taylor

**Affiliations:** 1 Public Partnership Group, School of Human & Health Sciences, University of Huddersfield, Huddersfield, UK; 2 Center for Applied Research in Health, School of Human & Health Sciences, University of Huddersfield, Huddersfield, UK; 3 Department of Health Sciences, University of York, London, UK

**Keywords:** Co-production, Public involvement, PPI, Participatory research, Research training service users and carers

## Abstract

**Background:**

As the role of Patient and Public Involvement contributors expands to all stages of the research cycle, there is increasing demand for training that meets the needs of this diverse population. To help meet this demand the National Institute for Health Research Collaboration for Leadership in Applied Health Research and Care, Yorkshire and Humber, worked with members of the public to develop a bespoke training package. The University of Huddersfield’s Public Partnership Group were invited to host the training and undertake an independent evaluation.

**Methods:**

Participatory action research was used to structure the evaluation, such that participants in the training and public members of the evaluation team were co-collaborators with a robust, significant and visible share in the process. This is evidenced by public team members’ roles in undertaking the majority of data gathering, including surveys, non-participant observation and interviews, and analysis, engaging in all reflective discussions, leading on producing a formal report and contributing significant sections of this paper.

The evaluation was approved by a University ethics panel.

Public involvement consisted of the 13 participants who received the training, and 3 of the 6 members of the evaluation team. Data collection took place between November 2017 and March 2018.

**Results:**

The evaluation found that participants understood more about the research process from attending the training, gaining greater confidence in their ability to volunteer to get involved. It also highlighted the difficulties of meeting the training needs of a diverse group with varying experiences and expectations. Skilful facilitation was needed to maintain pace, whilst engaging people with different levels of interest and knowledge. The management of the environment to maximise comfort and involvement was important. Early feedback to the delivery team enabled timely updating of the package.

Involvement in the evaluation was initially daunting for the three public members of the team, but hugely enjoyable and fulfilling, as well as enriching the process and outcomes. In particular, public involvement in the analysis and interpretation stages increased the authenticity of the evaluation findings.

**Conclusions:**

This evaluation validated the training package and demonstrated the value and impact of Public Involvement at all levels in research.

## Plain English summary

### Background

The National Institute for Health Research Collaboration for Leadership in Applied Health Research and Care Yorkshire and Humber, worked with members of the public to develop a training package for members of the public to understand health research, and the ways they can be involved in doing it.

They collaborated to design an enjoyable and informative experience and then asked a university Public Partnership Group to host, and independently evaluate the package.

### Method and design

The evaluation team included three service users/ carers and three academics. We obtained ethical approval from the University, and consent from the participants to gather data. We used surveys, interviews and observation to find out about the participants’ experience.

### Findings and recommendations

We found that the package was well designed, skilfully delivered, interesting and informative. All participants, despite different experience and expectations, felt they understood more about the research process and had greater confidence in their ability to volunteer to get involved.

Delivery was rushed at times and breaks were short because there was a lot of information. The venue affected how comfortable participants felt.

We recommend reducing or simplifying the material to allow a slower pace and more breaks, more time in introductions, different ways to gain group feedback, andtaking care to create a comfortable training environment. A text book, or manual containing the materials in detail, would be a valuable addition.

The inclusion of public researchers in the evaluation team changed the way work was managed and completed for the better. At times it was difficult. The public members needed to be assertive to get their views understood and the experienced researchers needed to allow others to lead.

### Conclusion

The training package was enjoyable and did increase the participants’ knowledge, understanding, skills and confidence. The experience of being involved with the evaluation was enriching for the team.

## Background

Research, particularly in the health field which preferences quantitative methods, has traditionally seen the patients and public that research is intended to benefit as remote from its design and delivery. There is a growing movement to challenge this view which is endorsed within the UK by Government funded organisations. For example, the National Institute for Health Research (NIHR) states:

“The suggestion that members of the public are ‘subjects’ or ‘silent partners’ in research is no longer a tenable position to maintain for any research organisation wishing to fund high quality research. Partnership, reciprocity and openness are now fundamental to how research is done and to the successful translation of research results into practice.”[10]

The focus above on funding indicates the growing trend for Patient and Public Involvement (PPI) to be embedded in any bid for monies. This parallels the imperative from *Liberating the NHS*: “no decision about me without me” [5]. Thus, any organisation offering health or social service, or undertaking research in these areas is challenged to fully engage its service users, or risk exclusion from funding.

Whilst user-led pressure groups and NHS activists may be heartened by this policy shift, the ‘active’ involvement in research, advocated by Involve [7] often proves hard to achieve. Oliver et al. [12] suggest ‘political mandate’ and the desire for improved quality of research are the primary aims of Involvement. Political mandate alone seems likely to lead to minimal, tokenistic involvement. However, Brett et al.’s [4] systematic review suggests growing evidence for impact of PPI at all stages of the research process. For example, helping with design, ensuring language is sensitive and enhancing data collection. In order to achieve this more engaged approach, moving from tokenistic involvement to fuller participation, consideration needs to be given to the preparation, training and support necessary for people to make an effective contribution. Brett et al. [4] advise:

“offering service user training in research methodology may help maximize the service user involvement and empower service users in their contributions to the design of the study, providing service users with the tools to discuss outcomes and formulate questions rather than limiting their involvement to accounts of their experiences” (p.641).

Within this spirit and in order to promote, support and improve the scope and amount of involvement of the public in health research, a public involvement team from the Mental Health and Comorbidities Theme (DIAMONDs Programme, http://www.diamonds.nihr.ac.uk/home) of the National Institute for Health Research Collaboration for Leadership in Applied Health Research and Care Yorkshire and Humber (NIHR CLAHRC YH), worked with researchers to develop a bespoke training package for mental health service users and carers. This package was then offered to the wider CLAHRC programme as a generic resource.

The NIHR CLAHRC YH team initially piloted this awareness training package, guided by best evidence to date and produced collaboratively with PPI members, which was delivered in a one-day event. Feedback from participants led to a restructure of the package and materials, creating a two-day training programme of information sharing, discussion and group work.

Before offering the restructured awareness training package the delivery team invited the collaboration of the University of Huddersfield Public Partnership Group (PPG) who hosted the training and designed and conducted a formal structured evaluation. The PPG is a funded group within the School of Human and Health Sciences, chaired and led by Service Users and Carers, with co-opted academic affiliations. It aims to influence health and social care development through direct involvement with education and research.

## Methods

### Aim

The aim of the evaluation was to find out if the training would increase the knowledge, understanding, skills and hence confidence of the participants regarding their involvement in the research process.

### Design

A Process Evaluation Methodology [9] was employed to explore implementation and participants’ responses to and interactions with the training, using a combination of qualitative and descriptive quantitative data. The principles of collaborative participatory research [8] underpinned the design of the training package and the evaluation.

Initial planning meetings were held between the NIHR CLAHRC YH’s training implementation team, and members of the PPG where they discussed and agreed on an approach. All parties agreed that the evaluation should be co-produced with the academic and service user/carer (SUC) members of PPG being equal partners. Three SUCs (one of whom was an administrator for the PPG) and three academic staff with experience of educational and health research formed the evaluation team. Although participants were known to the SUCs, they varied in their background, health state, literacy, health literacy, and research knowledge and experience. Their interests in research also varied. The training package evaluation team were independent from the NIHR CLAHRC YH implementing team but worked in close collaboration to ensure that the evaluation met expectations of the training’s agreed objectives.

Benchmarks for the training evaluation were identified through feedback from researchers and service users. The discussion led to a decision not to include any ‘testing’ of participants’ increased ability to engage in PPI. This was for two reasons: firstly, adding a before and after measurement of skills and abilities risked turning an event that what was intended to focus on awareness and confidence raising into an assessed course, which PPG members did not want, and would likely have changed the dynamic of the evaluation. Secondly, although all participants had the potential for involvement over the weeks following the training, there was no guarantee that this would be the case, so there was an uncontrollable variable. What it does include relates to the expectations SUCs had of a training event and the goals that health researchers thought could be achieved by a training event in terms of enhancing PPI. This in turn led to the creation of four sets of data gathering:

1. Pre-training survey: to be completed after signing consent forms and before the first session commenced.
2. Post-training survey: to be completed immediately after the end of the second session.
3. Teaching observation: to be completed by two non-participant observers and specifically including at least one SUC team member.
4. Individual interviews three months after training.

In addition, the training PowerPoint presentation was reviewed by a SUC evaluation team member who was not a participant or observer at the training event.

### Participants and recruitment

The training invitation went to an already known, purposeful sample of adult SUCs who:

- had some knowledge of the research process
- were interested and committed to being involved in the training
- were available on the two days of training.

Of the fifteen people who responded to the invitation to attend, thirteen attended on day one and were included as evaluation participants.

### The training package

The training days took place in November 2017 at the evaluation team’s host institution.

The day one venue was a small room situated at a part of the building that ensured the minimum of external interruption. Participants sat round two circular tables which meant that a few participants from each table had their backs to the screen, having to turn around to face the front during the lecture presentations. Though the room had air conditioning it became uncomfortably warm especially in the afternoon for the group seated further from the exit.

The day two venue was in a large room with the tables arranged in two u-shaped formations, allowing all participants to sit facing the screen throughout. The central heating was difficult to regulate and the room became uncomfortably cold towards the end of the day.

On both training days, the teaching resources included a large screen with power point slides, plus hand-outs, pens, post-it notes and writing material on the tables. Two members of the evaluation team quietly observed the teaching process on both training days by taking a back seat behind each group of participants.

Day one commenced with an opportunity for everyone to introduce themselves and then the presentation focused on each stage of the research cycle. Day two recapped understanding and focused on aspects of the research cycle where public involvement was likely. One session specifically focused on introducing research methods commonly used in health services research.

The presenter used a lecture technique followed by posing a question or by outlining a group work task relevant to the topic just covered. The presenter then joined, chaired and facilitated one group while the co-facilitator similarly worked with the other group, as well as keeping time.

At the end of each group work task, the presenter and co-facilitator summarised the group’s findings. Finally, the presenter orally combined the two sets of findings into one summary and moved back to the front to introduce the next topic.

### Data collection

#### Pre and post training surveys

These were designed and piloted by PPG members. The pre-training survey was completed by all participants after signing consent forms (*n* = 13) and before the first session commenced. The post training survey was completed immediately after the end of the second session (*n* = 12, one participant could not attend day two). Questions aimed to understand the hopes and expectations of participants, and their experience of the training. They included a question asking them to rate their level of confidence on a scale of 1–10 where 1 was low confidence and 10 was very confident. Whilst it is acknowledged that this self-rating of confidence is limited in its significance, a decision was made not to attempt to build in a measure of change, as this would introduce a perceived element of ‘testing’ of their ability, which might be stressful and would run counter to the aims of the training.

#### Teaching observation procedure and measurements

The teaching observation were undertaken by one SUC and one academic, focus was mainly on the flow and effectiveness of the training package, including:

- Course structure and delivery
- Content volume and quality/readability
- Pace and understanding/assimilation /checking understanding/ opportunity for asking questions/clarification/ areas needing further development
- Group work discussions/balance between interaction participation
- Effectiveness of the delivery/methods

#### Post training individual interviews

At around 3 months post-training, participants who had consented to being contacted were invited to an interview either by telephone, email or face to face. Two of the SUCs and one academic undertook the interviews. This was a structured interview, designed to mirror the questions and areas of interest raised in the earlier surveys, allowed the opportunity to discuss any areas of increased skills if this was appropriate and included an overall judgement of their perceived level of confidence (*n* = 11).

### Analysis and development of recommendations

Analysis was conducted in two phases: immediately after the training, and 3 months later after the follow-up structured interviews. Descriptive statistics were used to explore perceived levels of confidence for Public Involvement among participants before and after the training. The free text responses from the pre and post training surveys, structured interviews and notes from the teaching observations were analysed to identify themes [11].

The first phase of analysis was undertaken by the three core researchers ensuring inter-rater agreement. This analysis plus the review of training materials were the basis of a series of four iterative, reflective discussions which helped to ensure a collaborative approach, to ensure mutually derived findings and to provide early formative feedback to the delivery team and to formulate recommendations:

- Discussion meeting 1 was between the two teaching observers (one a SUC and one an academic) after the first training day to compare notes and review the observation method. This established a spirit of enquiry and debate that permeated the rest of the analysis. It affirmed the observation method and indicated areas for follow-up.
- Discussion meeting 2 included a second SUC who had reviewed the teaching materials, but had not been a participant or observer of the training. Each person had undertaken initial analysis and coding of the survey findings. Their analysis cross referenced with teaching observations and training materials review led to refined coding and initial theme identification.
- Discussion meeting 3 included the three people as above but now included a fourth SUC who had attended the training and completed the surveys, but was also a member of the evaluation team. Initial themes were scrutinised, debated and refined.
- Discussion meeting 4 was between the two teaching observers and the two delivery team members. This meeting allowed the evaluation team to give formative feedback to the delivery team to assist them in preparing for the roll-out of the training regionally. The two observers agreed to produce a reflective document prior to the meeting summarising the emergent findings, including tentative recommendations. The delivery team lead had also reflected from her perspective and these two documents formed the basis of the discussion. There was a strong congruence between the different perspectives, but also many points of debate.

Finally, following the three months gap between survey and interview data collection, coding and analysis of the structured interview data was conducted by the same analysts from phase one and incorporated into the findings. Two further discussion meetings between the evaluation team reviewed and consolidated the themes and recommendations.

## Results

### Pre- training survey findings

The pre-training survey feedback revealed a diverse group of 13 participants whose responses indicated a wide range of education levels and life experience.

The questions were intended to find out each participant’s individual hopes and expectations, their current involvement, if any, future aspirations after the training and how they currently rated their level of confidence in their involvement in health research.

The three key expectations revealed for the majority of participants were to gain more knowledge and information in general about health research; to understand research methods and process; and to understand the workings of the PPI field better.

The participants’ responses indicated that their expectations matched the training stated goals and objective. Two participants however reported having no specific expectations, adding that they hoped the training would address that.

Less frequently reported areas of interest were to learn more about consultation, co-production, collaboration, learning to navigate the system, addressing the ‘tick box’ culture, and working and dealing with staff in NHS organisations.

Potential areas of choice for future involvement identified by participants included:

- designing and/or evaluating research projects
- joining panels
- opportunity to sit in on student research training
- assisting students in choosing research topics for their dissertation
- getting more proactive in influencing services
- getting involved in more research work
- studying for further qualifications

### Post-survey training findings

The post training survey intended to find out whether the participants’ expectations had been met, what sort of things they had enjoyed, things they understood better, suggestions for change and their current level of confidence in involvement in future research.

Out of the 12 participants who were able to attend the second day, 10 reported that their expectations had been met, that their knowledge and information around PPI had improved and that they now had a better understanding of the research process. They also stated that they looked forward to more engagement and involvement. Two reported that though they had no set expectations, they had learned a lot.

All the participants reported that they enjoyed the group discussions most. Some enjoyed the informal atmosphere while others appreciated the way the delivery team was approachable, which made them feel welcome and at home.

Participants made a number of suggestions in terms of how the training package could be improved:

- the length of the training be shortened
- the training days could be brought closer
- slower introductions session and participants should have name badges
- better room arrangements so that everyone can see everyone else
- pace to slow down so that group discussions are not rushed
- participants to report their own group discussion findings

The main message was that participants had learned and understood a great deal more about the research process and had a better understanding of PPI.

### Teaching observation findings

The teaching observation exercise focused on five main aspects of the teaching session

- Course structure and delivery:

The sessions were very well delivered with clear audibility and a good communication style. The course content was of high quality and the presenter demonstrated a high degree of skill and knowledge in both content and facilitation techniques, with clear learning intentions.

b) Content, volume, quality and readability:

Although the content was of high quality, there was too much information and so the central messages were sometimes lost. Some slides were very busy and complex, while some were too small to see clearly making readability difficult for the participants.

c) Pace and understanding/assimilation:

Participants were given opportunities to ask questions and seek further clarifications but this was not taken up. The pace of the presentation was perceived to be speedy, and at times there was an observable need to finish off group work in a rush and move swiftly to the next topic and at such times, the opportunity for checking understanding and assimilation was left unexploited.

d) Group work discussions/balance between interaction participation:

Group work sessions were observed to be well facilitated. The discussions were all very lively and animated. However before answering the question posed or engaging with the given task, participants regularly required the questions/group work tasks to be repeated, rephrased and explained by the facilitators.

e) Effectiveness of the delivery/methods:

Some distraction of the group work discussions was observed as some participants became focused and overinvolved in discussing deficiencies and challenges related to PPI work or organisations. When this happened, the facilitators were observed to tactfully use facilitation techniques to successfully guide the discussion back to the topic under discussion. On both days, it was observed that participants’ sensitivities were respected at all times and due respect and consideration given to all contributions.

A supplementary issue through both days was the extent to which the ambiance of the room including lighting, heating and positioning of chairs, affected participants. There was no clear consensus, with the same temperature being considered too hot or too cold.

Although the observers felt that the central message was sometimes blurred, and there were inevitable diversions and distraction, overall, the objectives of the training were being addressed and met.

### Individual post training interview findings

Of the 12 participants who had completed the training, 11 took part in the individual follow-up interview by telephone, email or face to face. The interview was focused at establishing whether on reflection, the participants could give a snap shot of:

- whether and how the training had helped
- whether they had had a chance to use the new knowledge
- what else could be included
- overall feel of level of confidence in volunteering for involvement

In terms of main things learnt, most of the participants reiterated what they had reported in the post training survey, which was that the research awareness training was informative, very well structured and very well delivered and was beneficial, even for new starters.

*“In order to exert some real influence public involvement does need some understanding of the systems in which it operates” (participant 9)*

Participants also held that the group discussions made it possible for them to learn more about research process from others who had had different life experiences and perspectives which was beneficial. Two participants expressed surprise at the complexity of research. One participant expressed concern about the level of distraction, volume of particular participants and domination of group discussions.

Participants generally reported that the training had helped improve their levels of confidence and had increased their scope of involvement.

*“ … learning to formulate a topic [it] helps me with my presentation – I am not very good with writing, and people are probably not very happy with my pronunciation … . I am a more confident speaker now” (participant 8)*

One participant now had time to think about other relevant issues that were not included in the survey or discussions. On reflection, the participant revealed a great sense pride, of achievement and enthusiasm for the future. These revelations show that the individual interviews brought out fresh feedback on aspects the participants had felt comfortable and free to report in a one to one interview.

### Perceived levels of confidence

**Fig. 1:**
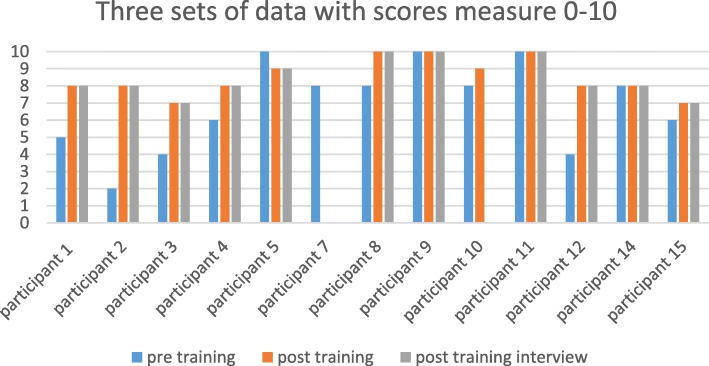
Reported Levels of Confidence. pre training (blue), post training (orange), post training interview (grey)

In the pre and post training surveys, and again during the interview, participants were asked to rate their level of confidence, from 0 to 10, 0 being no confidence and 10, feeling very confident: see Fig. 1.

Initially none of the participants perceived themselves as having no confidence. The lowest rating was two and the highest 10. The majority of participants felt their confidence had improved post training, and this confidence level appears not to have diminished over time.

## Discussion

The primary aim of the evaluation was to determine whether the training package increased the knowledge, understanding, skills and confidence of participants and to make recommendations regarding changes and improvements. This was achieved, with all participants taking new knowledge and confidence from the training, and the recommendations leading to an improved package that addressed the issues highlighted in the evaluation. This included taking greater care to securing an appropriate venue that offered physical and social comfort, which was identified as an important factor affecting engagement in the training in addition to the programme itself.

The discussion in this paper focuses on two areas of interest highlighted by the evaluation data and reflective discussions. Firstly, on the challenges of implementing PPI research awareness training and secondly on the insights gained regarding collaborative working between the public and academics from the training itself and the evaluation process.

### The challenge of implementation

One of the main items raised for discussion by the non-participant observers was the ways in which the main goal, despite being clearly articulated in the presentation, could be lost in the complexity of the content. This led to a reflection on the primary purpose of the training event: if it was, as understood, to help members of the public to understand the research process sufficiently to have the confidence to volunteer and contribute as part of a research process, then the theoretical content could be simplified to allow more time for their role to be foregrounded.

The informal conversations during breaks and group work plus analysis of the findings show a broad diversity in terms of age, sex, ethnicity, nature of service user/carer experience and educational and professional background. It is therefore unsurprising that pitching a standardised training package to them would be challenging: not dumbing down whilst avoiding jargon; allowing time to respectfully listen to individual participants’ views, without losing pace and focus; stretching and inspiring those who want challenge, without offending those who are just enjoying an interesting day out.

Fawcett et al. [6], suggest that people with experiential knowledge make valuable contributions in forums such as these, but that in turn means that each person wants their particular narrative heard. An anonymised example from the group work is of one participant having a pertinent experience of an issue and taking some time to share it. The facilitator, anxious to use the anecdote effectively employed skilful group work techniques to incorporate the information but also keep the pace moving. Findings from the evaluation include comments about group work being a little rushed, some participants dominating the discussion and some feeling they had not been given enough time to speak. All of these comments could legitimately relate to this single example.

The extent to which the different needs of individual participants could be accommodated whilst maintaining the integrity, pace and flow of the training was a significant consideration. The recommendation, to keep the intellectual level but prune some of the more detailed content to allow more time for participant interaction, was an attempt to address this. Supplementary materials, for example in the form of a manual, workbook or textbook, might be desirable to help those wanting more detailed information. Having a skilled facilitator for each group was also essential, and this has implications for course capacity and the resources required for future implementation. Alternatively, working with one smaller group could have helped with inclusiveness, although this has implications for future sustainability and capacity of training.

### The challenge of collaboration

In the early stages of planning the evaluation, the three evaluation team members who were leading the work (two SUCs and one academic) met to plan. It took the two SUCs an hour or more of determined, assertive dialogue to frame the evaluation into a format they understood and felt empowered to actively undertake. This does not reflect a lack of ability on the SUCs part, nor reluctance to engage from the academic. Rather, it shows how difficult it is to unpick the deeply embedded power imbalances that exist between researchers and SUCs and the different understandings about research that may contribute to this. The exchange led to a genuinely independent role for the SUCs, letting go of power by the academic, and a lot of learning.

Similar tensions can be seen in the dynamics of the training in the findings from the evaluation. There was no doubt that the training was sincere in its intention to empower participants by presenting the full picture of the research cycle and the places where they might be involved in it. However, the whole process remained firmly within a model where the research world invites the public to enter and become involved on its terms. The content of the training honestly acknowledged that PPI had a growing, and important role to play, but that it remained limited in its ability to frame the focus, design and delivery of actual funded research. The interactions and group work were respectful and warm, but tacitly reinforced the notion that the repository of power and knowledge rests with the research community.

Policy and practical guidance on PPI speaks positively of wanting genuine PPI involvement, with detailed guidance on why, and how to get involved (National Institute for Health Research). However, despite the espoused desire for authentic involvement, the requirement to comply with policy [12] remains far easier to achieve than equal partnership. The paradox in Beresford and Campbell’s [2] paper is contemporary despite its date: SUCs who have the knowledge, background and confidence to challenge those in authority can be dismissed as ‘unrepresentative’ and thus marginalised. In a research involvement context, the ‘expert by experience’ may only be valued for their personal experience of a particular health need, rather than welcomed as someone who could direct the research themselves. The Survivor Research Network manifesto [13] is one example of an alternative model of user-led research.

The difficulties of achieving authentic participation, when a consumer, or market led model of involvement is easier to achieve, and less likely to disrupt traditional power [1, 3], is apparent in the evaluation. However, offering high quality training that opens up the research process did raise the participants’ knowledge base and confidence to engage. Furthermore, the experience of undertaking this evaluation has demonstrated the possibilities of beginning to shift to a more collaborative position.

## Conclusions

The findings in this report underscore evidence that indicates that the PPI research awareness training package had indeed increased the knowledge, understanding and skills and that the participants’ level of confidence had been raised. The training had already enabled some participants to get involved in different and new areas of research processes, and the package has been updated in line with the evaluation recommendations, further demonstrating that training and evaluation objectives had been met.

The evaluation highlights the challenges of delivering high quality training to the diverse PPI population, and of offering the public truly democratic involvement. However, it also demonstrates that it is possible to successfully deliver high quality, academically challenging training to members of the public, and adds to increasing evidence of the added value of including PPI contributors as partners in research.

## Abbreviations

DoH: Department of Health
NHS: National Health Service
NIHR CLAHRC YH: National Institute for Health Research Collaboration for Leadership in Applied Health Research and Care Yorkshire and Humber
PPG: Public Partnership Group
PPI: Patient and Public Involvement
SUCs: Service users and carers
